# Case presentation: HIV late presenter with anaplastic large cell lymphoma

**DOI:** 10.1186/1471-2334-13-S1-P17

**Published:** 2013-12-16

**Authors:** Ramona Popescu, Adina Ilie, Rodica Bacruban, Ruxandra Moroti, Adrian Streinu-Cercel

**Affiliations:** 1 National Institute for Infectious Diseases “Prof. Dr. Matei Balş”, Bucharest, Romania; 2 Carol Davila University of Medicine and Pharmacy, Bucharest, Romania

## Background

We present the management of a 46 year-old male late presenter, diagnosed in June 2013 with HIV infection B3 and anaplastic large cell lymphoma.

## Case report

The patient presented to the hospital with fever, chills and pharyngitis. He revealed that during the past year he had had several occurrences of these symptoms which were treated ambulatory. He also had massive right side submandibular and axillary adenopathy which had been present on and off for the past 2 months.

At admission he was pancytopenic and he had important biological inflammatory syndrome. He tested positive for HIV with a viral load of 1,985,354 copies/mL and a CD4 count of 41 cells/mL, classified as B3. To determine the exact cause of the lymphadenopathy we performed a ganglia excision and a medullar puncture.

The analysis of the lymph node showed anaplastic large cell lymphoma (ALCL). This type of lymphoma represents <3% of AIDS related lymphomas; until 2010 only 37 cases of ALCL + AIDS being published.

At the present the patient is very compliant to antiretroviral therapy with good tolerability. The last evaluation showed an impressive increase in CD4 count. He was also referred to a hematology clinic for the treatment of lymphoma, where he received CHOP: cyclophosphamide, adriamycin, vincristine, prednisone.

## Conclusion

Our patient, a late presenter male, with symptoms shown during the past year, diagnosed with anaplastic large cell lymphoma, had a favorable evolution under HAART and CHOP therapy despite the poor prognosis.

